# A feasibility study of a Family Focused Treatment for Adolescents with Bipolar Disorder—the FAB study

**DOI:** 10.1186/s40814-015-0038-7

**Published:** 2015-12-07

**Authors:** Joanne Neely, David Miklowitz, Ann Le Couteur, Vicky Ryan, Luke Vale, Ruth McGovern, Aditya Sharma

**Affiliations:** 1 Institute of Neuroscience, Newcastle University, Henry Wellcome Building, The Medical School, Framlington Place, Newcastle Upon Tyne, NE2 4HH UK; 2 Division of Child and Adolescent Psychiatry, UCLA Semel Institute, David Geffen School of Medicine at UCLA, 760 Westwood Plaza Room 58-217, Los Angeles, CA 90024-1759 USA; 3 Institute of Health & Society, Newcastle University, Baddiley-Clark Building, Richardson Road, Newcastle Upon Tyne, NE2 4AX UK; 4 Complex Neurodevelopmental Disorders, Adolescent Bipolar Service, Walkergate Park Hospital, Benfield, Newcastle Upon Tyne, NE6 4QD UK

**Keywords:** Early onset, Bipolar disorder, Psychological intervention, Trial, FFT-A UK, Young people, Adolescents, FAB

## Abstract

**Background:**

The aim of this study is to examine the feasibility of a future definitive randomised controlled trial of Family Focused Treatment for Adolescents UK (FFT-A UK) in the management of early-onset bipolar disorder (EOBD) (under 18 years). The FFT-A has been evaluated in the USA to augment the pharmacological treatment of adolescents with bipolar disorder (BD). The FFT-A UK has been condensed to 16 sessions over approximately 6 months to be utilised within the UK National Health Service.

Research from the USA suggests that families experience high levels of distress, stress, burden and family disharmony when living with a young person who has BD. The FFT-A UK is a family-based approach designed to increase understanding of BD (psycho-education), improve communication and increase ability to problem-solve.

**Methods/Design:**

The trial will examine the feasibility of a randomised, parallel group, non-blinded design and the procedures of a subsequent definitive trial. Thirty-three young people with BD and their families will be recruited. Participants will complete measures at baseline, on completion of the 6-month treatment and again after a further 6 months. The self-report measures include the Warwick Edinburgh Mental Well-being Scale, The McMaster Family Assessment Device (FAD), Conflict Behaviour Questionnaire aka ‘Interaction Behaviour Questionnaire’, EuroQuol EQ-5D-3L and EQ-5D-Y. Primary outcomes will be rates of eligibility, recruitment and retention, estimates of the variability in the self-report measures and assessment of the intervention delivery in the study population. Participants’ qualitative views on the measures and intervention will be sought to confirm the acceptability of intervention and study design. The health economics component will establish how cost-effectiveness will be assessed in a future definitive trial.

**Discussion:**

The study will produce a full trial protocol and amendments to the FFT-A UK to inform a well-designed multi-centre randomised controlled trial (RCT) as an adjunct to pharmacotherapy in the management of EOBD.

**Trial registration:**

Current Controlled Trials ISRCTN59769322

## Background

Bipolar disorder (BD) is a chronic remitting and relapsing mood disorder, characterised by mood swings from ‘lows’ (depression) to ‘highs’ (mania/hypomania) with periods of relative stability in between. It is most frequently diagnosed in young adults (20–30 years), but recent research has shown that the onset of symptoms can occur earlier. Early-onset BD (EOBD) often co-occurs with other mental health and developmental disorders making the diagnosis and management more difficult [1–3].

EOBD defined for this study as onset before 18 years of age is associated with high rates of impairment in emotional, cognitive and social development [4]. The National Institute for Health and Clinical Excellence (NICE) bipolar guidelines note that the involvement of families is important in the management of young people with EOBD and recommend psychological interventions over 6 months in combination with psychopharmacology [5].

There are a few evidence-based psychotherapeutic treatment options for EOBD. A number of psychological treatments used with adults with BD have been adapted for use with affected adolescents. These include Interpersonal and Social Rhythm Therapy [6], Dialectical Behavioural Therapy [7] and Cognitive Behavioural Therapy (CBT) [8, 9]. These treatments have been effective in research done in the USA and have shown to delay the recurrence of episodes, stabilise symptoms and improve medication adherence. Other researchers have tailored therapy to include the family in conjunction with the young person. This research has shown that family-focused CBT is effective in treating children aged between 7 and 13 years with BD [10]. Furthermore, recent research has suggested that reducing parent stress and improving family functioning may be the most potent ingredient of the treatment [11]. Fristad and colleagues [12] have shown that a multi-family psycho-education group method is effective in improving symptoms and social and family functioning. Miklowitz et al. have adapted their manual-based family treatment for adults for use with adolescents (FFT-A) [13]. The results of a 2-year randomised controlled trial showed that those adolescents who received the 9-month, 21-session intervention had a more favourable and rapid recovery from their index depressive episode than patients in enhanced care (mean 10.2 weeks, 95 % CI 1.04–3.29, *p* = 0.04) [13]. A further study showed that FFT-A had a stronger effect for those who live in families with high emotional stress levels whereby adolescents with BD in high expressed emotion families showed greater reductions in both depressive and manic symptoms [14]. A more recent study showed that FFT-A together with pharmacotherapy proved no more effective than pharmacotherapy with brief psychotherapy in speeding up recovery or delaying recurrence of BD in adolescents. However, FFT-A was more effective than brief psychotherapy in reducing the severity of manic symptoms in year 2 [*F*(8,742) = 1.98, *p* = 0.046] [15].

BD affects not only the patient but also their family. Family-based interventions may therefore offer advantages in the long-term management of this disorder. One study has reported that there are high levels of stress in parents of youths with BD [16]. Reinares and colleagues [17] showed that a psycho-education family intervention reduced the distress and subjective burden and the caregiver’s belief about the link between the objective burden and the patient. Other studies in families with BD children acknowledged significantly more minor conflicts with family members than either unipolar or control group families [18]. Belardinelli et al. [19] found that the family environment of bipolar children in comparison to healthy children showed greater levels of dysfunction as measured by the Family Environment Scales. Parents of children with BD reported lower levels of family cohesion (*p* < 0.001), expressiveness (*p* = 0.005), active-recreational orientation (*p* < 0.001), intellectual-cultural orientation (*p* = 0.04) and higher levels of conflict (*p* < 0.001) than parents with no bipolar children. Furthermore, a study reported higher levels of conflict (*z*=4.729, *p* < 0.0001) on the Family Environment Scales in families where one parent had BD than families where neither parent has BD [20]. That family functioning is important in the prognostic outcome of EOBD has been demonstrated in a study that showed decreased family problem solving ability, as measured by the Family Assessment Device (FAD), predicted increased persistence of adolescents’ depressive symptoms [21]. The psychotherapeutic studies for EOBD to date have been undertaken in the USA. Given the differences in the health care systems between the USA and the UK, it is important to evaluate the effectiveness (including the cost-effectiveness) of psychotherapeutic evaluations for EOBD in the UK. This study intends to examine mental well-being and impact on family functioning along with quality of life adjusted years (QALYs).

### Aim

The aim of this study is to examine the feasibility of a future randomised controlled trial (RCT) of family-focused treatment (FFT-A UK) in the management of EOBD. The FFT-A was first adapted from the FFT in the USA to treat young people with BD and their families. The FFT-A UK version is further adapted from the original. These adaptations include some textual changes to make the version more relevant to UK culture (e.g. Coach replaced with Therapist) along with being condensed into 16 sessions taking place over 6 months (as opposed to the existing FFT-A version at the time of 21 sessions taking place over 9 months).

The study will investigate the acceptability of the FFT-A UK and explore issues of eligibility, randomisation, recruitment and retention rates and variability in the proposed outcome measures to inform the design of a multi-centre RCT with long-term follow-up.

The specific objectives of this feasibility trial are:

1. Is it feasible to deliver the FFT-A UK to young people (under 18 years) and their families?
2. What are the likely consent, eligibility and retention rates as well as the acceptability of being randomised to a delayed treatment arm?
3. What are service users and their family’s views about taking part in a RCT and completing the study assessments and outcome measures?
4. What is the variability in the outcome measures?

The results of this feasibility study will inform the development of a definitive multi-centre RCT to evaluate the clinical and cost-effectiveness of FFT-A UK as an adjunct to pharmacotherapy in the management of EOBD. Our hypothesis for the definitive RCT will be that the addition of a family-focused psycho-educational treatment will be more effective and cost-effective in the management of EOBD than treatment as usual (TAU) defined as review appointments every 3–6 months with their Consultant Psychiatrist.

## Methods/Design

### Design

The feasibility trial of a modified version of the FFT-A will examine the design and procedures for a subsequent definitive trial of the FFT-A UK for use in UK community-based settings. This is a mixed methods feasibility study with two components:

1. A single-centre, open randomised controlled trial of FFT-A UK in the management of EOBD compared to treatment as usual. The primary outcome measures are rates of recruitment, randomisation and data completion. We will also investigate the variability in the validated questionnaires to determine the best measures to use in a future definitive trial.
2. Qualitative interviews of a purposively sampled sub-set of families eligible for the feasibility trial to explore willingness to participate, be randomised and explore overall trial experience. An attempt will be made to undertake qualitative assessments with families who did not consent to be randomised.

### Participants

Thirty-three (to allow for a 10 % attrition rate) young people with a clinical diagnosis of EOBD and their families will be recruited into this trial.

### Inclusion criteria

1. Confirmation of diagnosis of BD and currently in remission using the WASH U KSADS (Washington University at St. Louis Kiddie Schedule for Affective Disorders and Schizophrenia) [22]
2. Fluent in the English language
3. Typically developing with ability in the average range and able to engage in psychotherapy (i.e. attending or having attended mainstream school)
4. Age between 11 and 18 years

### Exclusion criteria

1. Not meeting criteria for a diagnosis of BD according to the WASH U KSADS
2. Currently unwell in an episode of bipolar disorder (e.g. mania/hypomania/depression/mixed episode)
3. Lack of fluency in the English language that might prevent engagement in psychotherapy
4. Low intellectual functioning that might not allow young people to engage in the FFT-A UK

### Recruitment

Referrals were made through:

1. The National Tertiary Adolescent Bipolar Service, Northumberland Tyne and Wear NHS Foundation Trust.
2. Consultant Child and Adolescent Psychiatrists within Child and Adolescent Mental Health Services (CAMHS) in North East England. With the permission of the treating Consultant Child and Adolescent Psychiatrist, a Clinical Studies Officer (CSO) from the Clinical Research Network (CRN) will search patient records electronically and inform the clinician of potential participant families.
3. Advertising this study in the North East England with Bipolar UK, via their newsletter, ‘Pendulum’. Potential participants will be asked to contact their clinically responsible Consultant Child and Adolescent Psychiatrist to discuss the research project.

### Consent component 1

The referring Consultant Psychiatrist will give the young person and their family the Study Information Pack (SIP). The SIP will consist of information sheets for the young person, parents/carer and an Expression of Interest (EOI) form. The clinician will ask the family if he/she can forward their contact details to the research team so that they can ask more questions about the study before making a decision about giving written informed consent. Alternatively, using the contact information (email, telephone and/or post) on the EOI form, the research team can contact the young people and their families directly. Potential participants will be given at least 48 h to read the information sheets before the consent visit will take place. The research team will then request a CSO from the North East and North Cumbria CRN to set up a mutually convenient time and venue to meet with the young person and his/her family to further discuss this project in detail. Those wishing to take part are asked to provide written informed consent by initialling, signing and dating a study consent form which is witnessed by the CSO who has documented delegated responsibility to do so. Written informed consent is always obtained before any study-specific procedures including eligibility assessments and the collection of baseline data take place. Informed consent and assent are taken as appropriate. The original signed consent/assent forms are retained in the investigator site file with a copy retained in the clinical notes and another copy provided to the participant and family. Those families who decline to take part will be asked if they would like to give their reasons, though the right to refuse to participate without giving reasons is respected. After consent and assent, the eligibility assessments will be undertaken during a separate appointment.

### Consent component 2—qualitative interviews

Upon completion of the FFT-A UK, patients and their families will be invited to take part in the qualitative part of the study. They will be asked to comment about the treatment programme, fill out questionnaires, be randomised, etc. Information sheets will be given to families at the end of the final treatment session. After a minimum of 48 h, the research team will then request a CSO from the North East and North Cumbria CRN to set up a mutually convenient time and venue to meet with the young person and his/her family to further discuss this aspect of the project in detail. Those wishing to take part are asked to provide written informed consent by initialling, signing and dating a study consent form, which is witnessed by the CSO who has documented delegated responsibility to do so. Those consenting will be contacted by a member of the research team to discuss a suitable date, time and venue for the interview.

### Eligibility

Eligibility will be assessed using the WASH U KSADS [22]. This is a reliable and valid interview schedule used in research to confirm diagnosis and is carried out with the young person and his/her parent/carer. This eligibility assessment takes about 2–3 h to complete. Young people may participate if they meet the eligibility criteria and either are euthymic or have sub-threshold symptoms of an affective episode provided they are able to consent.

### Outcome measures

Most of the research studies on interventions in EOBD have focused their primary outcomes on clinical parameters such as symptomatic improvement, frequency of mood episodes and/or their duration, less need for hospitalisation or longer time spent in remission. Few studies have attempted to evaluate the intervention in terms of improvement in family functioning and/or health-related quality of life (HRQL). This mode of enquiry adopts a more patient-centred approach that focuses on the individuals’ perceptions of their physical, psychological and social functioning. An Australian study showed that children with mental health disorders were reported to have a worse HRQL than children with physical disorders and their problems interfered significantly with the daily lives of children, parents and family [23].

#### Validated quantitative questionnaires

##### The McMaster Family Assessment Device (FAD) [24]

The FAD is a 60-item, 4-point Likert-type questionnaire designed to evaluate families according to the McMaster Model of Family Functioning. It takes approximately 15 min to complete. It is made up of 6 scales which measure problem solving, communication, roles, affective responsiveness, affective involvement and behaviour control. In addition, there is a 12-item measure of general functioning that can be used as a global measure of family health/pathology [25]. It has good psychometric properties with good reliability and validity [26–28].

There are low correlations between FAD and the Marlowe-Crowne Social Desirability test, suggesting that the FAD responses are relatively free of the influence of social desirability [26]. This will be completed by parent/s.

##### Conflict Behaviour Questionnaire (CBQ) aka ‘Interaction Behaviour Questionnaire’ [29]

The CBQ is a self-report measure of problems with interpersonal behaviour between dyads (e.g. the mother about the adolescent or the adolescent about the father). Respondents answer true or false to questions about their relationship with a particular family member. The short form has 44 items and correlates 0.96 with the long version. It has been widely used [30, 31] and has adequate internal consistency and discriminant validity [29]. Both young people and one parent will complete this.

##### EuroQuol EQ-5D and EQ-5D-Y

EQ-5D is a standardised instrument for use as a measure of health outcome. The EQ-5D-Y is a youth version. It is developmentally appropriate and takes only a few minutes to complete. This will be completed by a young person and a parent. The EQ-5D descriptive system comprises 5 dimensions: mobility, self-care, usual activities, pain/discomfort and anxiety/depression. Each dimension has 3 levels: no problems, some problems, extreme problems. The EQ-5D-Y has the same basic system with some changes in wording. The EQ-5D is scored using UK population tariffs (a scoring system is under development for the EQ-5D-Y), which can be used in the calculation of quality adjusted life years (QALYs). QALYs are commonly used in economic evaluations.

##### Warwick Edinburgh Mental Well-being Scale (WEMWBS) [32]

This scale has been developed by Warwick and Edinburgh Universities to assess mental well-being. WEMWBS is a 14-item scale with 5 response categories, summed to provide a single score ranging from 14 to 70. The items are all worded positively and cover both feeling and functioning aspects of mental well-being. Its psychometric properties are robust and it is easy to complete [33]. This will be completed by the young person.

Self-completed baseline questionnaires will be given to the young person (3 in total, completion time, 30 min) and his/her family (3 for primary caregiver, completion time approx. 30 min), and an appointment for the research associate to collect these will be arranged. Assistance, via the CSO, will be offered to those who have difficulty filling them in. Once the questionnaires have been completed and returned, the randomisation procedure will occur. Randomisation will occur after baseline questionnaire completion as we do not want the type of treatment group allocation to bias questionnaire responses. Participants will be given a voucher (‘love to shop’) for £10 on completion of each batch of questionnaires (£30 in total). Participants and their families will complete these sets of questionnaires on 3 separate occasions, before randomisation, after treatment (at 6 months) and again after 12 months following randomisation.

### Randomisation

Participants will be randomised to ‘immediate treatment arm’ or ‘delayed treatment arm’ in a 1:1 ratio, using random permuted blocks. The randomisation allocation schedule will be generated by a statistician with no other involvement in the study. Randomisation will be performed by a trained member of the research team or CSO, using a secure password-protected Web-based system administered by Newcastle Clinical Trials Unit (https://apps.ncl.ac.uk/random). Randomisation will generate a unique 2-digit ‘study ID number’ for each participant.

### Interventions

#### Control condition: delayed treatment with treatment as usual

Families and the young person with bipolar disorder will receive usual care delivered by their referring Consultant Child and Adolescent Psychiatrist and the CAMHS team (usually a review appointment every 3–6 months). Families and young people will be offered the intervention after 1 year when all trial data collection is completed.

#### Intervention condition: immediate treatment

All family members living at home or involved in the care of the young person will be invited to attend the treatment and will receive 16 sessions of 1-h duration with a therapist over 25 weeks (1 therapist, 1 family unit). Treatment will be suspended if the young person becomes unwell as declared to the research team or Therapist by their referring Clinician.

The treatment structure includes:

1. Seven weekly psycho-education sessions. During these sessions, the family and young person will be given information about the aetiology, treatment and self-management of BD. A relapse prevention plan and identifying early prodromal signs to lessen the impact and possible need for hospitalisation will be considered.
2. Four fortnightly communication enhancement sessions will teach young people and their parents communication skills, offer positive feedback and active listening, make positive requests for change in others’ behaviour and give negative feedback.
3. Four fortnightly problem solving skill training sessions encourage an open dialogue between family members about difficult topics and help all involved to develop strategies for solving these problems.
4. A final overview session completes the intervention.

Participants will complete validated questionnaires, at baseline, immediately post intervention and again at follow-up after 6 months (T0, T6 and T12 months).

Treatment may take longer than 6 months if the young person becomes unwell. They will continue to get their therapy when they recover from an episode. If the T6 and T12 questionnaires are delayed by more than 6 months from the dates when they were meant to be completed, this will be considered as missing data. The young person and his/her family will be considered as a ‘dropout’ if he/she tells the research team that he/she no longer wishes to continue.

Families will be contacted by the research team on a monthly basis via text or email throughout the trial to be kept updated and to maintain good retention rates. On completion of the research study, young people will receive a certificate thanking them for their participation.

#### Training

All therapists will have undergone a 1-day workshop (9 am–5 pm) with Professor David Miklowitz, the author of the Family Focused Treatment—Adolescent (FFT-A). Each therapy session will be video-recorded, and 25 % of these will be viewed by David Miklowitz to ensure fidelity, using the Therapist Competence and Adherence Scale [34]. Therapists are given fortnightly supervision by AS, provided with written feedback and Skype supervision every 2 months by David Miklowitz.

### Participant compliance

Where feasible, appointments will be arranged at the participants’ convenience at a NHS venue of their choice. Therapists will arrange clinical appointments and record which family members attend each session. Should a participant become unwell during the study, his/her intervention will be ‘suspended’ and he/she will resume therapy when he/she is well enough to participate in the FFT-A UK as determined by their treating clinician. The primary outcomes for this feasibility trial are rates of eligibility, recruitment, intervention delivery and participant retention at follow-up. These will be recorded on case report forms by the Trial Coordinator.

### Economic evaluation

A review of the existing health economics databases will be conducted to identify possible alternative measures of health state utility with young people with EOBD and their families. The search will be conducted in the two major health economic evaluation databases: NHS Economic Evaluation Database (NHS EED) and the Cost-Effectiveness Analysis Registry. NHS EED includes structured abstracts of quality-assessed full economic evaluations of health care technologies, with the only criterion for inclusion in the database is that the study is a full economic evaluation. The economic evaluations are selected as a result of hand searching journals, health technology assessment reports and electronic searches of CINAHL, Embase, MEDLINE and PsycINFO. Abstracts are prepared based upon the potential relevance of the study to the United Kingdom National Health Service (UK NHS), which is important as all health-related quality of life outcomes for this study should ideally have been used in the UK NHS setting (or a closely comparable alternative).

Additionally, this review will also use the Cost-Effectiveness Analysis Registry. This registry focuses on a particular type of economic evaluation—a cost utility analysis. This form of analysis requires measures of health-related quality of life, so it may be applicable to our review.

As a result of the review, we hope to identify the following:

1. What tools have been used to gather health utility data in similar settings?
2. Where were these tools used and are they potentially suitable for use in a multi-centre definitive trial?
3. Key references from other researchers in the field if applicable to a UK NHS setting.

### Sample size considerations

No formal sample size calculation has been performed for this feasibility study as the primary outcome measures are concerned with the recruitment and randomisation to the trial and the acceptability of the trial in this population of patients. However, a sample size of *n* = 30 patients will provide sufficient data [35] to estimate the variability in the outcome measures at baseline and assess the feasibility of the trial. Data from a recent similar RfPB-funded study, ‘Beating Anxiety Together’ project from the NE of England [36], indicates that two thirds of families approached consented to taking part in the research and that attrition during the therapy phase was less than 10 %. Therefore, in order to achieve 30 participants who complete the trial and provide follow-up data, it may be necessary to approach 50 patients.

### Quantitative data and analysis

A database will be designed to facilitate accurate data entry with inbuilt checks, and a proportion of the data entry will be checked. As this is a feasibility study, the analyses of the data collected will be mainly descriptive, with 95 % confidence intervals reported where appropriate.

At baseline, the distribution of all numerical variables will be examined graphically and summarised by appropriate measures of location and spread. Similarly, baseline categorical variables will be tabulated and percentages reported. No formal statistical comparisons between treatment arms will be made; any comparisons will be descriptive and exploratory. Confidence limits for the estimated standard deviations of key study parameters will be calculated and used in sensitivity analyses for sample size calculations for a future multi-centre RCT study application.

### Qualitative data and analysis

Semi-structured interviews with young people and family members in the trial will be employed to gather their individual views in detail, while a focus group will allow trial participants to feed back on the findings from the individual interviews, and provide socially constructed perspectives to contrast with individual ones. Employing both methods will enable the participation of both those who may feel inhibited by group discussion and those unable or unwilling to attend interviews.

We will purposively sample families (*n* = 15), including three families in the control condition, seeking a representative male/female ratio and diversity in terms of age, diagnosis and time since diagnosis, family members and size. An attempt will be made to interview 2 family members within each family either face to face at the University, at home or by telephone by a qualitative researcher, giving a sample of 30 participants. Interviews will commence after 6 months following the last session of the intervention, with each interview expected to last approximately 45 min. Since it will also be important to feasibility to know why families may not wish to take part in the intervention or research, we will also invite a member from each of 5 families refusing participation to consent to a brief telephone interview on a voluntary basis to raise concerns they may have had. The interview agendas will initially be developed by members of the Project Advisory Group (PAG) including our parent and young people representatives to cover areas of research interest (attitudes towards and experiences of both the intervention and research procedures). The agenda will be flexible to allow both expected and emergent themes to be incorporated into later interviews [37].

**Fig. 1:**
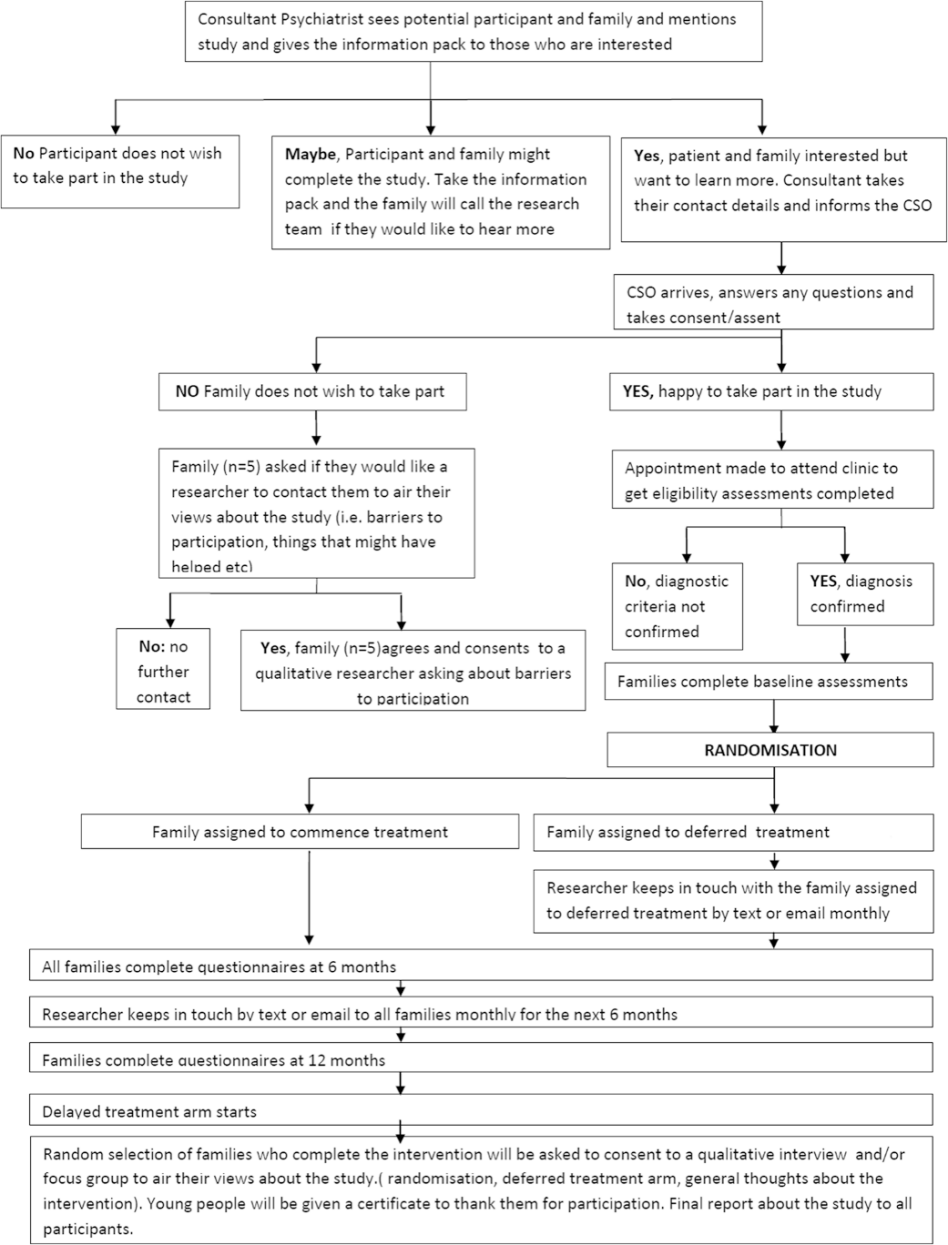
Study protocol flowchart

Both interviewees and family members who were not interviewed will be invited to a focus group intended to provide feedback on the initial findings and gain the families’ perspectives on the outcomes of analysis. The focus group discussion will be structured around a presentation of the initial findings, thus allowing both interviewees and non-interviewees with comparable circumstances to comment on how these represent the experience and attitudes of families affected by BD in general and increase confidence in the broader applicability of findings [38]. Interviews and the focus group will be tape-recorded, with participant’s written consent, transcribed, anonymised and subjected to framework analysis [39]. This is an appropriate approach for qualitative health research with objectives linked to quantitative investigation [40]. We will use NVivo to aid indexing and charting. The data will be repeatedly read and coded independently by two researchers to increase the reliability of the study. When discrepancies between coders exist, these will be discussed until a consensus is reached. Analysis will be discussed at regular meetings of the research team in order to identify areas for closer consideration (including negative case analysis) and to enhance credibility of the thematic framework and interpretation [38]. Figure 1 shows a flow diagram from the feasibility study.

### Ethical and research governance approval

The study has been granted a favourable ethical opinion by Sunderland NHS Research Ethics Committee (13/NE/0117).

### Project timetable

The trial duration is 36 months. Recruitment commenced on 15th January 2015 and is ongoing.

## Discussion

Findings from this feasibility study will indicate whether and how a definitive trial can establish the effectiveness and cost-effectiveness of a family-focused treatment in the management of EOBD. The outcomes will include the protocol for such a trial, with a sample size calculation, which can usefully enhance the evidence base for psychological interventions in the treatment of EOBD. To the best of our knowledge, there are no trials in the UK to support evidence-based decisions for psychological treatments in the management of EOBD. The FFT-A has been developed in the USA by David Miklowitz, as a 9-month, 21-session psychological treatment. This study has condensed the sessions to 16 over a period of 6 months to try to ensure its applicability and acceptance to the NHS. It could be that the intervention requires further amendments or alterations. Findings from this study will determine if this is required.

Despite recommendations from NICE about involving families in the management of EOBD, there are no UK-based studies to inform the delivery of effective evidence-based interventions for the treatment of young people with EOBD and their families. This intervention, the FFT-A UK, is expected to improve family functioning and benefit HRQL. It is also expected to lessen the impact and severity of further episodes through teaching young people and their family’s skills to self-manage their chronic condition. Patient empowerment through self-management in a collaborative partnership is important for patient health and well-being [41, 42]. This project should heighten awareness amongst clinicians about the disorder which will encourage better recognition, earlier diagnosis and intervention (though not directly assessed in this trial). This in turn should improve the prognosis of EOBD by delaying and reducing the severity of relapse episodes and cutting costs in the longer term.

We plan to proceed to a full multi-centre RCT to determine the clinical effectiveness and cost-effectiveness of this intervention. If effective and cost-effective, we would hope that in keeping with NICE guidelines, the FFT-A UK will be an integral part of the assessment and management pathway for young people with bipolar disorder.

### Trial status

The trial is currently recruiting.
